# Conditional cash transfers to retain rural Kenyan women in the continuum of care during pregnancy, birth and the postnatal period: protocol for a cluster randomized controlled trial

**DOI:** 10.1186/s13063-019-3224-8

**Published:** 2019-03-01

**Authors:** Caroline A. Ochieng, Hassan Haghparast-Bidgoli, Neha Batura, Aloyce Odhiambo, Geordan Shannon, Andrew Copas, Tom Palmer, Sarah Dickin, Stacey Noel, Matthew Fielding, Sangoro Onyango, Sarah Odera, Alie Eleveld, Alex Mwaki, Fedra Vanhuyse, Jolene Skordis

**Affiliations:** 1 Stockholm Environment Institute, Linnegatan 87D, Box 24218, 10451 Stockholm, Sweden; 2 UCL Institute for Global Health, 3rd floor, Institute of Child Health, 30 Guilford Street, London, WC1N 1EH UK; 3 Safe Water and AIDS Project (SWAP), P.O. Box 3323, Kisumu, 40100 Kenya

**Keywords:** Conditional cash transfers, Maternal and child health, Antenatal care, Facility delivery, Postnatal care, Child immunization, Cluster randomized controlled trial, Kenya

## Abstract

**Background:**

Antenatal care (ANC), facility delivery and postnatal care (PNC) are proven to reduce maternal and child mortality and morbidity in high-burden settings. However, few pregnant rural women use these services sufficiently. This study aims to assess the impact, cost-effectiveness and scalability of conditional cash transfers to promote increased contact between pregnant women or women who have recently given birth and the formal healthcare system in Kenya.

**Methods:**

The intervention tested is a conditional cash transfer to women for ANC health visits, a facility birth and PNC visits until their newborn baby reaches 1 year of age. The study is a cluster randomized controlled trial in Siaya County, Kenya. The trial clusters are 48 randomly selected public primary health facilities, 24 of which are in the intervention arm of the study and 24 in the control arm. The unit of randomization is the health facility. A target sample of 7200 study participants comprises pregnant women identified and recruited at their first ANC visit over a 12-month recruitment period and their subsequent newborns. All pregnant women attending one of the selected trial facilities for their first ANC visit during the recruitment period are eligible for the trial and invited to participate. Enrolled mothers are followed up at all health visits during their pregnancy, at facility delivery and for a number of visits after delivery. They are also contacted at three additional time points after enrolling in the study: 5–10days after enrolment, 6 months after the expected delivery date and 12 27 months after birth. If they have not delivered in a facility, there is an additional follow-up 2 wees after the expected due date. The impact of the conditional cash transfers on maternal healthcare services and utilization will be measured by the trial’s primary outcomes: the proportion of all eligible ANC visits made during pregnancy, delivery at a health facility, the proportion of all eligible PNC visits attended, the proportion of referrals attended during the pregnancy and the postnatal period, and the proportion of eligible child immunization appointments attended. Secondary outcomes include; health screening and infection control, live birth, maternal and child survival 48 h after delivery, exclusive breastfeeding, post-partum contraceptive use and maternal and newborn morbidity. Data sources for the measurement of outcomes include routine health records, an electronic card-reader system and telephone surveys and focus group discussions. A full economic evaluation will be conducted to assess the cost of delivery and cost effectiveness of the intervention and the benefit incidence and equity impact of trial activities and outcomes.

**Discussion:**

This trial will contribute to evidence on the effectiveness and cost-effectiveness of conditional cash transfers in facilitating health visits and promoting maternal and child health in rural Kenya and in other comparable contexts.

**Trial registration:**

ClinicalTrials.gov, NCT03021070. Registered on 13 January 2017.

**Electronic supplementary material:**

The online version of this article (10.1186/s13063-019-3224-8) contains supplementary material, which is available to authorized users.

## Background

A “Continuum of Care” for reproductive, maternal, newborn and child health (RMNCH) includes integrated service delivery for mothers and children from pre-pregnancy to delivery, the immediate postnatal period and early childhood [1]. Assuring continuity of care has become a key programme strategy for improving the health of mothers and newborns, and is an important measure of service quality for outcomes such as prevention of mother-to-child transmission of HIV (PMTCT) [2]. Yet, in many countries, there remain major gaps in seeking care along this continuum. Retaining women in this continuum even during pregnancy has not yet been achieved in sub-Saharan Africa (SSA), with only 44% of women attending the minimum of four recommended visits that constitute focused antenatal care (ANC) [3]. Focused ANC is a scheduled service, and when clients do not adhere to the recommended timing or frequency, the effectiveness of the service is compromised. A number of complementary treatments provided during ANC have to be delivered in sequence for them to be effective. Examples include iron and folic acid supplementation, tetanus toxoid immunization, syphilis testing and treatment, counselling on maternal and infant nutrition and intermittent preventive treatment of malaria in pregnancy (IPTp) [4]. Postnatal services are equally essential, as half of all postnatal maternal deaths occur during the first week after delivery, and one in four child deaths occur in the first month of life [5]. In SSA, postnatal care programmes are among the weakest of all reproductive and child health programmes [6]. Follow-up treatment and services such as PMTCT, family planning, breastfeeding and infant feeding support and childhood immunization cannot be achieved if women are lost to care immediately after delivery, as is the current trend.

Complex structural, financial, cultural and behavioural factors contribute to gaps in care seeking in SSA [7, 8]. In Kenya, where maternal mortality rates are 488 per 100,000 live births, a lack of transport has been cited as one of the major contributing factors to low utilization of healthcare services [9]. In a national survey [9], the largest proportion of respondents (42%) reported that delivering outside a health facility took place because the facility was far away or they lacked transport to the facility. In comparison, only 17% cited the fees levied at the facilities as the key barrier [9]. Other barriers include the indirect costs of care seeking such as food for mothers and accompanying children, new clothes appropriate to be seen at ANC visits, and the opportunity cost of time away from farming or other income-generating activities [10]. Cultural barriers have also been reported. In Zimbabwe, Chapman [11] found that because women have high maternal morbidity and miscarriages in their communities, and at the same time are under high social pressure to bear children, they hide their pregnancies to protect themselves and the unborn child from witchcraft and sorcery. Lack of knowledge about the benefits of care seeking is yet another important barrier [10]. One study in Kenya showed that the motivation to attend ANC was driven by authority-linked factors, such as obtaining an ANC card to avoid reprimand or reduced care by the staff during birth [10]. The visits are therefore made late in pregnancy, and discontinued once the ANC card is obtained. In the light of these multiple challenges, an intervention targeted at only one of the barriers (e.g. removal of user fees, or provision of transport vouchers) will only have a limited impact.

Personal financial incentives (PFIs) may offer the broad-based solution needed to tackle low levels of care seeking and retention of women in the care continuum. PFIs can tackle both the financial barriers to care seeking and the motivational barriers. Several studies [12–15] have demonstrated how personal financial incentives (PFIs) can help to increase adherence to lengthy treatment schedules. In developed countries, paying people to change their behaviour has been effective in reducing excess consumption of high-fat foods, cigarettes, alcohol and illicit drugs [16]. Notable successes in developing countries include tuberculosis (TB) treatment programmes, where financial and material incentives directed to patients increased successful completion of TB treatment regimens [17, 18]. Outside the context of TB, PFI schemes in SSA remain largely untested. In the context of reproductive, maternal, neonatal and child health, few cash transfer programmes have been piloted, and none have targeted adherence to a scheduled programme or treatment schedule; instead the focus has been on a specific aspect of the care system such as ANC attendance or facility delivery [19, 20].

There is an opportunity to test the effectiveness of PFI as a broad-based intervention to improve maternal care-seeking behavior in the Kenyan context. Over two thirds of women come into contact with the formal healthcare system at least once during pregnancy [3]. A strategy that can retain these women in care, from their first contact in pregnancy through to delivery and the childhood period, could significantly alter the negative trends in maternal and child health in this context and possibly also in Africa more generally.

This trial is testing the effectiveness and cost-effectiveness of cash incentives in retaining women in the continuum of care from their first ANC visit until their children reach 1 year of age. It will be the first trial in SSA targeted at retaining women in the maternal, newborn and child health (MNCH) care continuum, and will generate crucial evidence to inform policy and practice. A secondary aim of the project is to assess the benefits of improved care-seeking in maternal and child health, and the cost-effectiveness of the intervention.

## Methods

### Study setting

The study is conducted in Siaya County, Kenya, located on the shores of Lake Victoria, in the West of the country. Siaya County has a population of 984,069 people and is made up of six sub-counties, which are mostly rural and perform poorly on several development and health indicators [21]. As a whole, the County has a human development index (HDI) score of 0.46, significantly below the national average of 0.56 [22]. It has the highest national rates of HIV infection, TB and malaria in Kenya, and among the worst indicators of child and overall health status [23]. Infant mortality stands at 111 per 1000 live births against 49/1000 for the country, and maternal mortality at 695 per 100,000 live births against 488/100,000 in Kenya. On a few indicators, the County performs better than the national average. For instance, 38% of the population in Siaya live below the poverty line, while the country’s average is 45% [24]. The rate of immunization in children under 1 year is 72.5% against 67.5% for the country, and the rate of delivery by a skilled attendant is 69.6% against a 61.2% national average [23].

In 2015, Siaya County had 174 health facilities with 123 categorized as public, 7 as non-governmental, 16 as faith-based and 28 as private [25]. Health facilities in Kenya are graded into 6 levels. Level 1 facilities are found at the community level, facilitate community diagnosis, management and referral to upper facility levels and encourage appropriate healthy behaviours. Level 2 facilities, known as Dispensaries, are the interface between the community and the health system, and offer basic curative, case management, prevention and promotion services and basic ANC. Level 3 Health Centres offer curative and case management services for infectious and chronic illnesses and inpatient care, and may cover a catchment of 10,000 people or more. Level 4 facilities are sub-County Hospitals that serve as secondary care facilities but also offer primary healthcare services such as ANC. Level 5 facilities are County Referral Hospitals and offer a broader spectrum of specialized services. Finally, the level 6 facility is a National Referral Hospital, Kenyatta Hospital in Nairobi [26]. The health facilities in Siaya County include one level 5 hospital and one level 4 facility in each sub-County [27]. The rest are mainly level 3 Health Centres and level 2 Dispensaries staffed by nurses or clinical officers, and level 1 community facilities staffed by community health volunteers (CHVs). Overall, there is low coverage of healthcare in the County, with a physician-to-population ratio of 1: 62,000, and nurse-to-population ratio of 1: 2500 [25].

The implementing project partner, Safe Water and AIDS Project (SWAP- http://www.swapkenya.org/), has been working in the region since 2005 to improve maternal and child health. Their work has focused on level 1 services, which are aimed at empowering Kenyan households and communities to take charge of improving their own health, and fall within the Kenya Essential Package for Health (KEPH) Community Strategy. They work in close collaboration with the Ministry of Health and Siaya County Government. Despite these and several other governmental, non-governmental organization (NGO) and donor funded efforts at community level, there still remains a wide gap in reaching pregnant women with the essential package of services that formal healthcare facilities would routinely deliver to those retained in the continuum of care.

### Trial design

The study is a cluster randomized controlled trial (cRCT) in which 24 clusters are randomized to receive the intervention and 24 clusters are randomized to the control.

#### Cluster size and selection

Level 2 or 3 health facilities are the units of randomization. Level 2 or 3 facilities are comparable and comprise Dispensaries and Health Centres, respectively. While there may be some difference in the services provided at these facilities, only those offering the full profile of antenatal services were considered for inclusion in the study. All eligible clients attending participating facilities during the enrolment period were recruited into the trial, subject to consent. Based on the background data on ANC attendance in the study region, it is expected that each of the 48 clusters will recruit 150 participants into the trial, giving a total sample of 7200 eligible women.

#### Randomization

A master list of 84 eligible level 2 and 3 health facilities in Siaya County was generated in December 2016. The master list was stratified by sub-County, so that selection onto the master list was proportional to the number of facilities in each sub-County. From the stratified master list, a short-list of 60 facilities was randomly selected for potential inclusion in the study. The random selection of the 60 short-listed facilities was thus also stratified by sub-County, with the shortlist in each sub-County proportional to the number of eligible health facilities on the master list for that sub-County. Facilities were selected so that catchment areas did not overlap.

In January 2017, the 60 shortlisted facilities were reduced to a final sample of 48 facilities for inclusion in the trial, 24 in the intervention and 24 in the control arm. The selection of the 48 final facilities included in the trial, and their allocation to intervention or control, took place in a public forum to ensure that the selection and allocation processes were transparent and acceptable to senior management in the County Government. To achieve this level of transparency in the selection process, a public meeting was organized. The County Director and the Medical officer of health (MoH) in charge of each of the six sub-counties in Siaya were invited to attend the meeting and participate in the health facility selection and mapping. Each MoH was asked to bring along the County Records Officer (CRO) and another member of their sub-County Health Management Team (CHMT).

During this meeting, the names of all 60 short-listed facilities were written on individual pieces of paper, folded and placed into six transparent plastic boxes - one for each sub-County. The total target sample of 48 facilities was divided between sub-Counties broadly in proportion to the number of facilities in the master list, but with rounding so that each sub-County had an even number of facilities in the trial. The Health Management Teams from each sub-County were invited in turn to select a piece of paper from their respective box, beginning with the Northernmost sub-County. The first facility selected was allocated to the intervention arm and the second to the control arm, the third to intervention and so on in succession. The name of each selected facility was written on a board, together with the allocation. For each selected facility, the records officer for the sub-county was asked to map the location and catchment area on a large map of the county. If a subsequently selected facility was found to have an overlapping catchment area with a facility previously selected, then the newly selected facility was rejected and another drawn from the box to take its place. This process continued until 48 facilities had been selected and allocated for the trial. This meeting generated a list of 24 intervention and 24 control facilities, with their location and catchment areas mapped to ensure that they do not overlap. In summary, the randomization is stratified by sub-County and ensures equal allocation to study arms within each stratum.

#### Trial participants

Eligible participants are women attending their first ANC visit in public primary healthcare facilities in the study area. The ANC attendance rate for at least one visit is very high in Kenya, and more than 80% in Siaya County [10]. The majority of visits take place at level 2 Public Dispensaries and level 3 Health Centers, where the government has abolished user fees for maternal care since June 2014 [28]. Participant recruitment will not be conducted in private clinics, which levy user fees and are more likely to be visited by a small number of women of higher socio-economic status. Participants will also not be recruited from level 4 or 5 facilities, as this would bias the sample towards more complicated pregnancies or serious health conditions.

Trained and incentivized health staff determine if a pregnant woman meets the study eligibility criteria by administering screening questions at the end of her ANC visit. Criteria for enrolment are:

i. Women attending their first ANC visit;
ii. Long-term resident of the catchment area served by the health facility, with long-term residence defined as living in the area for at least 6 months; and
iii. Women with access to a mobile phone that belongs either to themselves or to a member of their household or person whom they trust. Access to a mobile phone is a project requirement for the cash transfer payments and participant follow up. This requirement was easily met by all women (*n* = 200) during the pilot phase of this project.

If a woman is eligible, the recruiting staff explain the objectives of the study and seek her consent to participate.

**Fig. 1:**
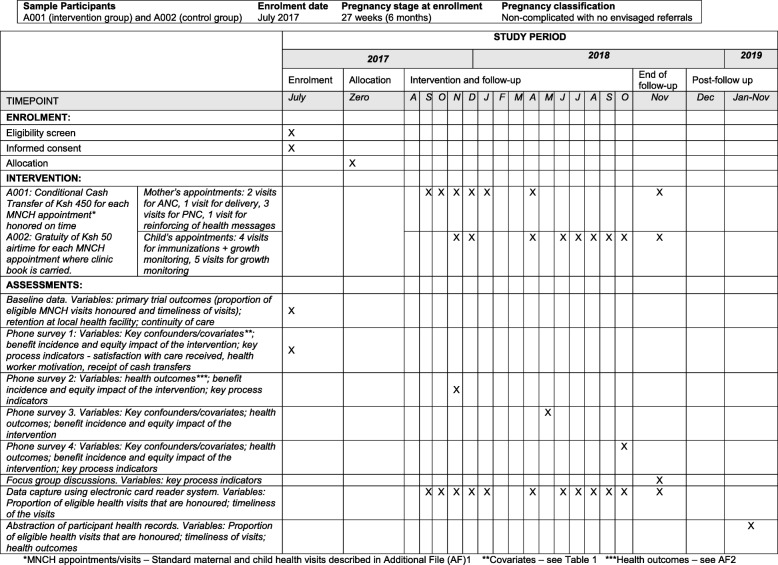
Schedule of enrolment, interventions and assessments. *MNCH appointments/visits – Standard maternal and child health visits described in Additional file 1. **Covariates - see Table 1. ***Health outcomes - see Additional file 2. MNCH, maternal, newborn and child health; ANC, antenatal care; PNC, postnatal care

Enrolment into the study commenced in June 2017 (Fig. 1) and is ongoing. Initially, the enrolment duration was estimated to be 9 months, with an estimated 816 pregnant women recruited into the study each month, leading to a total sample of 7200 women. However, a nationwide health workers’ strike began in June 2017 shortly after initiation of the study and continued until November 2017. During this period, 18 health facilities continued to provide health services with the support of NGOs, and were able to enrol participants. The remaining 30 health facilities were not operational and only started to enrol participants in November 2017 after the strike had been resolved. As of February 2018, the project has enrolled 2841 pregnant women. At the time of writing, enrolment is expected to proceed until July 2018 or until the sample size of 7200 is reached.

Once enrolled into the study, the participant is followed 341 up until the expected baby is 1 year of age (Additional file 1). Participants who move out of the study location are considered lost to follow up.

### Intervention strategy

#### Cash transfer value

The intervention is a conditional cash transfer (CCT) payment for each facility appointment attended for ANC, delivery, postnatal care and childhood immunization; and referrals related to any of these visits. For each verified health visit that is made on time following enrolment, a woman receives a cash transfer of Ksh 450 (4.5 USD). The value reduces by one quarter for each week of delayed visit, and no reward is offered for any visit made 3 weeks after the appointment date. On average, Ksh 450 equates to the transportation cost of a local taxi to the health facility, and is a value that has been approved by the research ethics committee for many studies in the region as reasonable compensation for research participants.

#### Card-reader system for automatic tracking of health visits and cash transfers

##### *Afya* card

Once consent to participate in the trial is obtained, all women recruited into the study are given an ANC clinic book as is standard practice. Additionally, they are provided with a special enrolment card (*Afya* card) that contains their study ID and that is linked to a card reader terminal installed at all participating health facilities. The card is attached to the clinic book so that participants do not leave it behind when making the health visits.

The *Afya* card is the size of a credit card and stores holder data. These include authentication information (study ID, study arm, clinic at which enrolled), pregnancy-related information (pregnancy stage at enrolment, expected delivery date (EDD), parity) and information on health visits made (where and when) throughout the study period. The background and pregnancy-related information is collected at enrolment by a nurse at the clinic, who then loads it onto the card upon touching the card to the reader. The nurse also keys in the date of enrolment and the date of the next visit, which gets loaded onto the card upon touching the reader. Subsequent health visits by participants are recorded in their clinic books and the clinic records as is standard practice (Additional file 2). Additionally, the visits are automatically tracked by touching the card on the card reader. At the end of each visit, the nurse keys into the reader the date of the next health visit, to enable automatic tracking of whether the visit is attended and how timely the visit was.

##### Card reader

Card readers are installed in all participating intervention and control facilities. The device used is a Famoco Fx100, an android near field communication (NFC) card reader. The Fx100 has an in-built SIM card slot and is enabled for GSM/GPRS/3G communication. The card reader is connected to a remote server that electronically personalizes the NFC card of the user during enrolment, and tracks her subsequent health visits. Each time the *Afya* card makes contact with the reader, the information stored on the card is transmitted to the reader and from the reader to the card, then subsequently from the reader to the web portal (an electronic database) [29]. The card also has an offline transacting capability with the terminal reader that enables it to function during occasional absence of connectivity. The reader also comes with a power back up that enables it to function when there is no electricity for a prolonged duration.

This web portal enables the study team to remotely track participants’ health facility attendance throughout pregnancy and up until 12 months after delivery, and to effect the cash transfer payments. On confirmation of a timely visit, payments to participants are made directly from the project’s bank account where the funds are held, to the participants’ mobile money accounts. This is achieved through integration of the Famaco FX card reader system with Safaricom Bulk Disbursement Service, the leading mobile money payment provider in Kenya. A payout is thus automatically triggered following the touching of the *Afya* card on any of the 48 terminal readers. Manual payouts are triggered by the project team upon verification of visits by participants who forget to carry their clinic books during the visits or who visit non-enrolling facilities that have no readers installed. Pregnant women are therefore not restricted to using the facility at which they were enrolled as long as it is within the study evaluation zone, which is Siaya County. Health visits outside Siaya County are not recorded or rewarded, except for high-risk groups (see Collecting data on visits to facilities outside the study area).

The web portal is continually monitored by a member of the project team to ensure that there are no pending payments for timely health visits attended. The project team also makes regular visits to the health facilities to monitor whether all system components function as expected, and to resolve any challenges promptly.

#### Gratuity for control group participants

Although the primary function of the cash transfer is to motivate health facility visits, the cash payment could additionally motivate women in the intervention group to carry their ANC clinic books when they visit the clinics. This is because the *Afya* card embedded in the book facilitates automated payment transfers to the women at the end of the clinic visit. Forgetting to carry the ANC clinic card during ANC visits delays the payment as the visit must be verified manually. Therefore, to balance out the possible health effects of carrying the ANC clinic book in the intervention and control groups, a nominal gratuity for carrying the clinic book is offered to women in the control group, who might have lower motivation to carry the book. The gratuity is in the form of mobile phone airtime with a value of Ksh 50 (0.5 USD) for every eligible visit, transferred through the same system that is used to issue the incentives. Ksh.50 is approximately equivalent to the cost of five bananas purchased at a market and would not compensate participants for the cost of attending the health visit. It may however, be an incentive for a woman who was going to go to the clinic anyway, to carry her clinic book.

Participants in both study groups are followed up in the same way, including monitoring of visits using the electronic-based system and telephone surveys conducted at key points in the care continuum. Follow up is described Data collection methods.

#### Health staff incentives

Trained and incentivized health staff determine if a pregnant woman meets the study eligibility criteria by administering screening questions at the end of her ANC visit. Health staff also collect background information on participants and key it into the card reader. During subsequent visits, staff key in the next visit dates on the readers to enable electronic tracking of whether the visit was attended and when it was attended. This determines the amount of cash incentive the participant receives. Staff are paid 400 Ksh (about 4 USD) per person enrolled into the trial, to recognize their time and effort in enrolling participants and tracking subsequent health visits.

#### Theory of change

**Fig. 2:**
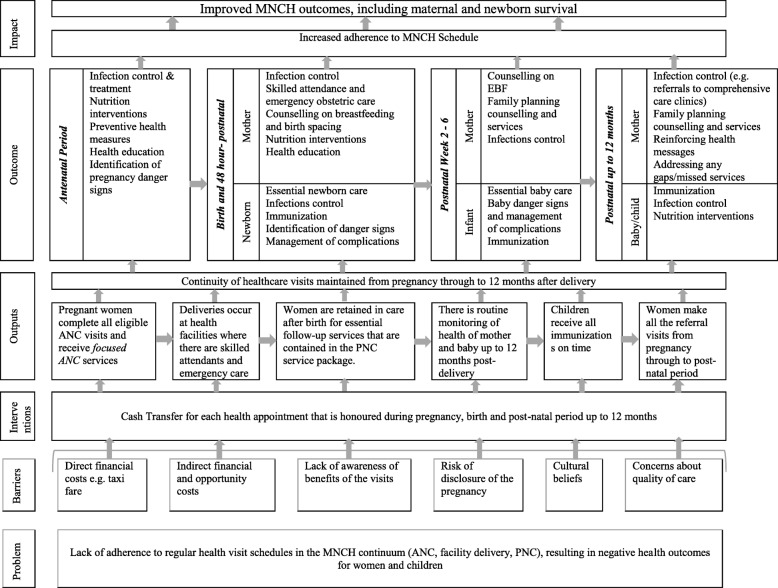
Theory of change. MNCH, maternal, newborn and child health; ANC, antenatal care; PNC, postnatal care; EBF, Exclusively breastfeeding

The intervention’s theory of change [30] is described in Fig. 2 and is informed by the World Health Organization (WHO) guidelines for health visit schedules for women during pregnancy, birth and the postnatal period [4]. Extensive evidence supports the health benefits of services offered during these visits [4] (Additional file 4).

The rationale for the use of a CCT intervention is found in behavioural economic theory, which suggests that individuals commonly have inconsistent preferences for similar outcomes occurring at different points in the future, with outcomes in the near future generally valued more than those in the distant future [31, 32]. According to Giles et al. [33], while the health gains of health-promoting behaviours are often delayed in time, the financial and opportunity costs are usually immediate. Individuals thus make a rational choice to pursue unhealthy behaviours or delay spending on health-promoting activities in the present but with future benefits. Cash transfers work by bringing long-term goals into the short-term decision horizon by providing immediate rewards and reducing current costs [33].

This study is based on the theory that cash transfers overcome direct and indirect financial and behavioural barriers to healthcare attendance during pregnancy, delivery and the postnatal period, and in so doing would lead to more regular use of services compared to current practice. Retention of women in the care continuum from pregnancy through the postnatal period will have a positive impact for MNCH interventions that require regular treatment and follow up.

### Research questions

This study aims to investigate whether CCTs are effective in retaining women in the continuum of care for MNCH. The secondary objectives of the study aim to establish whether regular care-seeking within the MNCH continuum, incentivized by the CCTs, leads to improved maternal and child health outcomes and whether it can be cost-effective. To answer these questions, a number of primary and secondary outcomes will be measured.

#### Primary outcomes

The following primary outcomes will be measured and compared between the two study arms:

1. The proportion of eligible ANC visits made after recruitment
2. The proportion of participants delivering babies at a health facility
3. The proportion of eligible health appointments attended for PNC
4. The proportion of expected immunization appointments attended by children
5. The proportion of health referrals attended for ANC, PNC and child immunization

#### Secondary outcomes

The secondary outcomes are:

1. Proportion of women retained in the continuum of MNCH from pregnancy to 12 months post-delivery
2. Likelihood of a live birth and child survival 48 h after birth
3. Likelihood of maternal death during delivery and 48 h postpartum
4. Self-rated health of mothers at 6 months and 12 months after delivery
5. Mother’s perception of infant’s health 6 months and 12 months after delivery
6. Proportion of mothers exclusively breastfeeding to 6 months of age
7. Proportion of women using family planning or contraceptives after delivery
8. Screening and control of infections for mothers and fetus/baby during pregnancy and postnatal periods
9. Cost of delivery and cost effectiveness of the intervention
10. Benefit incidence and equity impact of the intervention

Process evaluation [34] will document the design, implementation and mechanisms of the intervention. A full economic evaluation will be conducted to assess the cost of delivery and cost effectiveness of the intervention and the benefit incidence and equity impact of trial activities and outcomes.

### Sample size

We hope to analyse all the primary outcomes jointly and also to analyse most outcomes as ordinal variables (see Data management and analysis), thereby maximising power and aiding interpretation. However, for our sample size calculation, we consider power to detect an effect of the intervention on one primary outcome, which we also assume to be a binary indicator that all attendances are made, as this is simple and conservative in the power achieved. The expected prevalence of these indicators in the control arm ranges between 30 and 80%. Little specific information on the likely intra cluster correlation (ICC) for any of the outcomes is currently available - we consider it may lie between 0.005 (low) and 0.025 (moderate). Our planned sample size is 48 clusters (24 per arm) and an average cluster size of 150. At a low ICC, the design effect (DE) will be 1.745 and hence the effective sample size (ESS) will be 2063 participants per study arm. At a moderate ICC, the DE will be 4.725 and hence the ESS will be 762 per arm. Power to detect absolute differences is lowest when the prevalence is 50% and highest when the prevalence is either high (towards 100%) or low (towards 0%). Here we consider the prevalence in the control arm to range between 50% (‘worst-case scenario’) and 80% (‘best-case scenario’). We consider the standard 5% significance level. If the prevalence of the outcome is 50% in the control arm, the sample size provides 80% power to detect an improvement to 54.5% in the intervention arm if the ICC is low and 57.5% if the ICC is moderate. If the prevalence of the outcome is 80% in the control arm, the sample size provides 80% power to detect an improvement to 83.5% if the ICC is low and 85.5% if the ICC is moderate.

### Data collection methods

Data are collected in person by nurses at enrolment, over the telephone by trained interviewers at key time points, and through manual extraction of routine health records supplemented by the electronic contact card system. Data are also collected to facilitate rigorous process and economic evaluations. Table 1 summarizes the data collected using each method.

**Table 1:** Primary and secondary outcomes of the trial and sources of these data for different outcomes

Primary outcomes	Source of data
• Proportion of all eligible ANC visits made after recruitment • Delivery at a health facility • The proportion of health appointments honoured for PNC at week 2 to 6 after delivery, and months 4–12 after delivery • Proportion of referrals for ANC, PNC and child immunization attended Proportions of immunization appointments attended by children	• Abstracted health records from the health facility • Electronic system used to enrol participants into the study • Telephone surveys will collect visits made outside the study area by all “high risk” women and 50% of other women
Secondary outcomes	Source of data
• Proportion of women retained in the continuum of MNCH from pregnancy to 12 months post-delivery	• Clinic records • Electronic card system • Telephone survey 3 at 6 months after delivery
• Likelihood of a live birth and child survival 48 h after birth	• Clinic records • Telephone survey 2 at 2 weeks after EDD, if not delivered at the facility
• Likelihood of a maternal death during delivery and 48 h postpartum	• Clinic records • Telephone survey 2, 2 weeks after EDD, if not delivered at the facility
• Self-rated health of mothers, 6 months and 12 months after delivery	• Telephone surveys 1, 3 and 4 at 6 months and 12 months after delivery
• Mother’s perception of infant’s health, 6 months and 12 months after delivery	• Telephone surveys 2, 3 and 4 at 6 months and 12 months after delivery
• Proportion of mothers exclusively breastfeeding to 6 months of age	• Telephone surveys 2 and 3 and 4 at 6 months and 12 months after delivery
• Proportion of women using family planning and contraceptive use after delivery	• Telephone surveys #3 and #4 at 6 months and 12 months after delivery
• Screening and control of infections for mothers and fetus/baby during pregnancy and postnatal periods	• Clinic records (ANC, HF deliveries and PNC clinic records) • Manual collection of health records
• Cost of delivery and cost effectiveness of the intervention	• Project accounts • Key informant interviews • Facility costing tool
• Benefit incidence and equity impact of the intervention	• Clinic records • Electronic system • Telephone surveys 1, 2, 3, 4
Key confounders/covariates	Source of data
• Expected date of delivery • Education • Asset ownership • Age • Distance to facility • Level of health facility • Marital status • Number of children • Planned pregnancy • Quality of care (e.g. waiting time) • Migration away from study area	• Enrolment survey at health facility • Clinic records • Telephone survey 1, 3, 4
Key process indicators	Source of data
• Satisfaction with care received	• Telephone surveys 1, 2 and 3 at 2 weeks, 6 months and 12 months after delivery, focus group discussions with a sub-sample of participating women in the intervention area
• Health worker motivation	• Key informant interviews with health service providers
• Receipt of the *Afya* and/or any other transfers	• Telephone surveys 3 and 4 at 6 months and 12 months after delivery

*MNCH* maternal, newborn and child health, *ANC* antenatal care, *PNC* postnatal care, *EDD* expected delivery date

#### Data collection at enrolment

During enrolment, after written informed consent has been obtained, a trained nurse at the enrolling facility extracts background information from the participant’s health records and enters them into the card reader as described in section Card-reader system for automatic tracking of health visits and cash transfers. The extracted data include name, age, marital status, parity, stage of pregnancy, expected date of delivery (EDD), area of residence and mobile phone number.

#### Telephone surveys

Data are collected on secondary outcomes, socio-economic characteristics, care seeking and decision-making regarding the cash transfer, using the four follow-up telephone surveys described subsequently. Some data on the primary outcomes are also collected (see Table 1). The surveys are administered to all participants in both the intervention and control groups.

Telephone survey one: after enrolment

The first telephone interview is conducted with all participants as soon as possible after enrolment. This structured questionnaire collects data on socio-economic status, self-rated health and decision-making. Information on the recent health visit is also collected, including cost and time taken to travel to the facility, waiting time, cost of all care received and satisfaction with the care received. The same questionnaire is administered to the intervention and control groups, and the interviewer is blinded to the allocation of the participant.

Telephone survey two: 2 weeks after the EDD, if no facility delivery

A phone survey is conducted with all mothers who delivered babies at home, 2 weeks after their EDD. This survey collects information on delivery date, delivery outcome, who conducted the delivery, self-rated wellness for mother and child and breastfeeding initiation. The interviewer is blind to participant allocation.

Telephone survey three: 6 months after the EDD/child birth

A telephone survey is administered to 50% of participants 6 months after the EDD, to collect data on secondary outcomes such as maternal and child deaths, self-rated health of mothers and infants, exclusive breastfeeding and utilization of family planning methods. This survey also collects information on the last health care visit including the cost of seeking care, purpose of visit, waiting time and visit experience.

A second part of the survey is used to elicit information on the receipt of any conditional or unconditional cash transfer, receipt of *Afya* transfers (in the intervention arm only), how any money received was spent and who decided how the money was spent. The interviewer is not blind to participant allocation, as some questions are related to receipt and utilization of the cash transfers.

Telephone survey four: 12 months after the actual delivery date

A final survey is conducted with the same 50% of participants who completed telephone survey three. This survey again collects data on secondary outcomes such as maternal and child deaths, self-rated health, breastfeeding and utilization of family planning methods, information on the latest health care visit, and receipt and use of any cash transfers. Moreover, this survey collects additional information on complementary feeding and immunization. The interviewer is blind to participant allocation except for the final part that seeks information on receipt of cash transfers and its usage.

#### Manual extraction of health records

At the end of the enrolment period and throughout the remaining trial period, health seeking and health status data will be manually extracted from routine health records held at facilities. Additional file 2 shows the detailed health records that are taken during ANC, delivery and postnatal health visits. A record book is also available at health facilities for each child, and a child growth card is issued to women who deliver at health facilities. This card contains key data on the newborn from birth until they reach 5 years of age.

Towards the end of the trial period, data on health visits and services will also be collected from referral facilities attended by the participants. Data on visits made to health facilities outside Siaya County will not be extracted or rewarded. Participants will be identified in the records by their study ID number^1^ to ensure participant confidentiality.

#### Electronic capture of health visits

A secondary method for collecting data on the primary health outcomes is the electronic card reading system as described previously (see “Card reader”). The system has been designed for the project by an information and communications technology (ICT) company in Kenya called Nailab. The system is fully owned by the project and will only be utilized to fulfill the objectives of the project. Using two primary data collection methods will enable us to triangulate our data and increase confidence in the trial results.

#### Collecting data on visits to facilities outside the study area

The strategies used to collect these data vary according to whether or not the participant is considered high risk. High-risk groups in this study comprise mothers whose ANC records show HIV-positive status, positive malaria smear test and/or other diagnoses of sexually transmitted infection (STI). These diagnoses are likely to require referrals and additional health visits not captured in routine ANC or PNC records. Furthermore, participants with these complications are more likely to be lost to the routine monitoring systems as they might completely shift their ANC, delivery and PNC visits to the referral centers that handle these infections.

We will track all health visits made by high-risk participants only, including those taking place in neighbouring counties outside the evaluation zone. This would be done through extra phone calls to the participants to elicit information on health facilities visited. The study team will then visit those facilities and manually extract health records as described previously. The exception to this case would be if care is sought outside the Western Kenya region, in which case the data will be considered missing in the case of a single instance of care seeking, or the participant will be classed as lost to follow up if they have permanently transferred.

For lower risk participants, we shall incorporate questions relating to the primary outcomes (facility visits, child vaccinations etc.) into the telephone surveys (see Telephone surveys) administered to 50% of participants. This gives participants the opportunity to report visits both outside the study area and within, and we shall also ask whether the participant moved temporarily or permanently outside the study area. By comparing these self-reports with the data on visits within the study area (collected by other means) we will be able to approximately deduce the number of visits made outside the study area. These additional visits will not be verified by the study team as will be done for high-risk participants.

#### Baseline data

In order to understand the baseline characteristics and behaviour of the sample population, a retrospective audit of ANC, delivery and PNC will be undertaken by chart review at selected facilities. The retrospective chart audit entails collection of data from all facilities, between January and December 2016 in the year preceding the commencement of participant enrolment. The data were collected according to a pre-determined proforma. This included data on primary trial outcomes, retention at the local facility and continuity of care. Data were anonymised at collection and each new entry was assigned a unique retrospective *Afya* identifier number.

#### Process evaluation

To inform replication and scale up of the intervention, a mixed method process evaluation is underway to document the design, implementation and mechanisms of the intervention. The process evaluation is aimed at answering the following questions:

1. What factors affect the intervention’s delivery and impact?
2. What are the likely operational requirements for delivering the intervention at scale?
3. How acceptable is the intervention to recipients?
4. What is the impact of the intervention on health staff motivation and job satisfaction?

Quantitative data for the process evaluation are being collected from health records, telephone survey data and *Afya* contact-card data as described previously for the main trial outcomes. Qualitative data for the process evaluation are being collected via key informant interviews with a sub-sample of participating health facility staff, focus group discussions with a sub-sample of participating women in the study area, with sampling to saturation and field notes from intervention team members and notes from the monthly debriefing meeting.

It is also expected that the intervention will increase demand for health services. However, no expansion in health service supply is planned and as such, the increase in demand will likely increase the workload of existing staff. To capture any unanticipated impact on effort, data on health workers’ motivation and satisfaction [35] will be collected in both arms of the trial. A less motivated health worker may perform adequately in minimally demanding conditions, but their willingness to exert extra effort might depend on whether they feel there is personal value in doing so [36]. Further, if a workplace is not conducive to effective working, or if health workers do not have the knowledge, skills or experience to complete their tasks, they might be demotivated. Thus, health worker motivation is the ability and willingness to put in the effort required to do the job, influenced by individual factors (such as knowledge, skills, experiences, psychological attributes) and organisational factors (such as physical and social environment, policies and practices) [37]. Organisational factors are more likely to change than individual personality tendencies, or societal and cultural values. Thus, with the introduction of the card system and the potential increase in the demand for MNCH services, health worker motivation is likely to be affected, with consequences for service delivery. A self-completion motivation and job satisfaction survey will thus be administered to all participating health staff. Collecting and analysing these data will allow us to explore how the intervention could affect health worker motivation and satisfaction [38], and potentially service delivery.

#### Economic evaluation

An economic evaluation will be conducted from the provider perspective. Costs incurred by implementing agencies will be collected prospectively from the project accounts and input into a customised Excel-based costing tool. Data on healthcare provider costs will be collected retrospectively from a random sample of participating health facilities in the control and intervention arms. Data on resource use for maternal and child services, including equipment, drugs, floor space, overheads and the time input of health facility staff, will be collected using key informant interviews with healthcare providers and financial controllers [39–41]. These data will be supplemented with information on participants’ health-seeking behaviour and utilization, collected through the card reader and follow-up phone surveys.

All costs will be adjusted for inflation using the Kenyan Consumer Price Index (CPI) and presented in international dollars. The incremental cost-effectiveness of the *Afya* intervention will be compared with the status quo alternative. Incremental cost-effectiveness ratios (ICERs) will be calculated for all statistically significant primary trial outcomes, as well as selected significant secondary outcomes. In addition to ICERs, results will be presented as a cost-consequence analysis [42, 43], listing all costs and outcomes separately, allowing policymakers to compare the costs and impacts of the *Afya* intervention.

### Data management and analysis

#### Blinding

Due to the nature of the intervention, participants are not blinded to their study allocation. Some data collection is, however, blinded to the intervention allocation status of participants as described in the data collection section. Project managers are not blinded to the allocation status of the study participants; however, the Principal Investigators and analysts will remain blinded. Data will be analysed blinded to the intervention status of participants.

#### Interim analysis and monitoring by the Data Management Committee (DMC)

We do not expect any adverse effects of the intervention but plan to carry out two interim analyses in 2018 and 2019, which will be reported to an independent Data Management Committee (DMC) to be convened according to the DAMOCLES charter [44]. For these meetings, the DMC will be provided with a report on the two key safety measures for the trial, presented separately by trial arm. The two key safety measures are infant and maternal mortality. The DMC will decide at each meeting whether to request further analyses, which may include analysis of the study primary outcomes, and will on each occasion recommend the study should either continue or stop. The DMC may also comment on whether any changes to the trial should be made, for example if the basis of the sample size calculation is contradicted by the accumulating data or if changes are required to improve recruitment or data capture. Only the trial statistician and the DMC will be aware of the outcomes on these safety measures.

#### Final analysis

Final analyses will be by intention-to-treat, based on all enrolled mothers who were pregnant during the recruitment period (July 2017 to July 2018) and their children, regardless of whether they received the intervention or not. We will test for differences in the primary outcomes between the intervention and control arms using logistic regression for binary outcomes, and ordinal logistic regression for ordinal outcomes, adjusting for clustering using random effect models.

Provided there is no qualitative difference between the odds ratios for the effect of the intervention across the primary outcomes, then only the summary odds ratio will also be presented. This will be the primary effect measure for the trial. It will be estimated using independence estimating equations because not all participants will be eligible for all outcomes (e.g. vaccination of infants who died) and this approach avoids any implicit imputation of outcomes for ineligible participants.

Besides the issue of ineligible outcomes described previously, there should be no completely missing data on the primary outcomes. If a record of attendance cannot be found in the patient records in the study area, this will be interpreted as failure to attend. However, participants may make visits to facilities outside the study area, often in relation to temporarily or permanently moving to reside outside the study area. As described in Section Collecting data on visits to facilities outside the study area, data on visits outside the study area will be available for all higher-risk participants, and dependent on telephone interviews also available (though approximate) for up to half the lower-risk participants.

We plan to conduct our primary analysis based only on data on lower-risk participants from visits within the study area if it seems this will not lead to bias, but to use the approximate number of visits outside the study area from (up to) half the lower-risk participants to impute these additional visit data for the remaining participants if it seems bias will otherwise arise. We prefer not to impute visits out of the study area unless this is required because the data on visits outside the area by lower-risk participants are only approximate.

The precise criteria by which will we judge if bias is likely, and exactly how imputation will be conducted if necessary, will be specified in our analysis plan. If imputation is conducted then the analysis based on imputed data will be considered primary.

In our primary analysis we will adjust for key predictors of the primary outcomes, and any other baseline factors of clusters or individual participants for which an important imbalance is seen between arms. We will also carry out sub-group analyses to examine whether the effect of the intervention differs by wealth/multi-dimensional poverty quintile to understand the equity impact of the intervention.

The final analysis will be presented according to the consolidated standards of reporting trials (CONSORT) requirements for cRCTs [45, 46]. Both the interim and final analyses will be conducted by the trial statisticians. A detailed statistical analysis plan will be prepared and finalized in consultation with the Trial Steering Committee (TSC)/DMC before analysis begins.

## Discussion

The ongoing intervention seeks to positively impact maternal and child healthcare-seeking in one of the poorest provinces in Kenya, improving survival and reducing morbidity among the most vulnerable. It aims to achieve this by enhancing the demand for continuity of care from the antenatal period through to the postnatal and early life period, thereby maximising the gains obtained from each stage in these processes. Being amongst the first rigorous evaluations of maternal health incentive payments in East Africa, it will generate results of great policy interest for the region and sub-Saharan Africa as a whole. In addition to providing evidence on the role of conditional cash transfers in encouraging uptake of maternal and child healthcare visits, it will also provide evidence on the benefit of an extended continuum of care on child survival and maternal health.

Both a strength and limitation of this trial design is the reliance on functioning technical systems to track health service use and make the cash transfers. In the rural Kenyan context, electricity provision may be unreliable and mobile phone coverage inconsistent. This may pose a risk to trial protocol adherence and manual back-up systems have been devised in the event of technical failure. A further limitation of the design is the follow up of a limited sub-sample of participants for the telephone interviews. While this is the most pragmatic and cost-effective design for the trial, a larger sample size would provide us with greater power to detect smaller effects.

It is further acknowledged, that this study addresses only the demand-side factors that negatively affect timely and repeated care-seeking in this context. Supply-side factors affecting care-seeking and health outcomes may reduce the observed effectiveness of the intervention. For example, it is well-documented that poor service quality and negative staff attitudes are also among the factors that keep women away from the health facilities [10]. The process evaluation previously described will assist in measuring the likely effect of supply-side factors on trial outcomes. Similarly, patient perceptions of quality of care will be collected during the telephone interviews. In addition, a sample of key informant interviews will be conducted with healthcare providers to explore provider perceptions of quality of care since the introduction of the scheme and their perception of whether the “carrying capacity” of their institution is being exceeded. It is likely however, that this will remain an area for future study.

## Trial status

The trial is ongoing. Recruitment began on 15 July 2017 and ended on 31 July 2018. Protocol Version 2.1, 26 December 2018.

## Additional files

Additional file 1: Rewarded health visits in the *Afya* project. (DOCX 27 kb)

Additional file 2: *Afya* secondary data. Information collected on all women who make facility visits in Siaya County, Kenya. (DOCX 36 kb)

Additional file 3: Annex 11a: Participant’s information sheet part 1, English. (DOCX 14 kb)

Additional file 4: Annex 13a: Informed consent form, English. Annex 14a: Consent to access medical records, English. (ZIP 23 kb)

## Abbreviations

ANC: Antenatal care
cCRT: Cluster randomized controlled trial
CCT: Conditional cash transfer
CHMT: County health management team
CHVs: Community health volunteers
CPI: Consumer price index
CRO: County records officer
DMC: Data management committee
EDD: Expected delivery date
HDI: Human development index
ICC: Intra cluster correlation
ICERs: Incremental cost-effectiveness ratios
IPTp: Intermittent preventive treatment of malaria in pregnant women
KEPH: Kenya Essential Package for Health
MNCH: Maternal, newborn and child health
MOH: Medical officer of health
NFC: Near field communication
NICE: National Institute for Health and Care Excellence
PFI: Personal financial incentives
PMTCT: Prevention of mother-to-child transmission
PNC: Postnatal care
RMNCH: Reproductive, maternal, newborn and child health
SSA: Sub-Saharan Africa
SWAP: Safe Water and AIDS Project
TB: Tuberculosis
TSC: Trial steering committee
WHO: World Health Organization

## Footnotes

1 The health records are maintained in a book whereby all the details of an individual are filled in one row in the data book, while the columns contain the data fields. In one of the blank columns, the nurse will add a removable sticker that contains the ID of each participant at enrolment (during the pilot they used a different pen colour for research participants; however, this is permanent and therefore not appropriate as the records have multiple uses). At the time of data extraction, a nurse will place a removable sticker on the column that contains the names of the participants, thereby concealing those names from the staff extracting the records. Participant ID numbers will however remain. The records will be extracted in clinic by taking a picture of the page on the book. Subsequent input of these data is described in see (Manual extraction of health records).
