# Supplementary material for: Conditional cash transfers to retain rural Kenyan women in the continuum of care during pregnancy, birth and the postnatal period: protocol for a cluster randomized controlled trial
Source: Trials. 2019 Mar 1;20:152. doi: 10.1186/s13063-019-3224-8 (PMC6397480; doi:10.1186/s13063-019-3224-8)
Supplement: Supplementary file 1 — Rewarded health visits in the Afya project. (DOCX 27 kb) [file 13063_2019_3224_MOESM1_ESM.docx]

**Additional file 1: Rewarded Health visits in Afya project**

**a: Maternal health visits that are standard health services in the continuum of care of MNCH**

| **Antenatal Period** | **Month 0 – 3**  **(1st visit at 8-12 weeks)** | | **Month 3 – 6**  **(2nd visit at 24-28 weeks)** | | **Month 6 – 9 (3rd visit at 32 weeks; 4^th^ visit at 36-38 weeks)** | **IMPACT** |
| --- | --- | --- | --- | --- | --- | --- |
| Services | Medical history, preventive measures (education; tetanus toxoid;iron&folate), screening tests (HIV, Syphillis), treatment for infections (malaria), identification of warning signs & 4-stage classification | | Follow-up, re-classification if needed, examinations (Anaemia, BP,fetal growth&movements), screening tests and treatments (e.g. ARV), reinforcement of health education, emergency birth plan | | Follow-up on previous visits: examination, screening tests, treatments if eligible, preventive measures, education on postnatal care, infant feeding,child spacing | Early identification of complications and treatment of infections = 37-67% lives saved in Africa, at 90% service coverage |
| **Birth and 48-hour postnatal** | **Month 9 - Birth** | | **Month 9 – 48 hour postnatal** | | |  |
| Services Mother | Skilled attendance and emergency obstetric care  Prevent and manage problems e.g. multiple births, postmaturity, abnormal positions of the baby (breech)  Prevent heammorhage, sepsis | | Focused physical exam, Maternal danger signs and management of complications; Counseling on exclusive breastfeeding (EBF) and LAM contraception; Healthy Timing and Spacing of Pregnancy (HTSP messages); Maternal nutrition interventions (iron and folic acid); HIV&syphilis tests as indicated, Referral to Comprehensive Care; Health education (e.g. use of ITNs) | | | M - Reduce 50% deaths that occur within one day of childbirth; Reduce 30% stillbirths that occur during labour;  Counselling impact: |
| Services Newborn | EBF, Essential newborn care; Incubators for pre-terms, Newborn physical exam, Newborn danger signs and management of complications, prevent asphyxia,infections,disability, Nevirapine as indicated; BCG and Hep B immunisation | | | | | N- Avert 30-50% deaths occuring on the first day of life |
| **Postnatal** | **Month 10 (2^nd^ week postnatal)** | **Month 11 (6^th^ week postnatal)** | | **Month 13 – 15 (4 – 6 months postnatal)** | **Month 19 - 22** |  |
| Maternal Services | EBF counseling, Education on Return to sexual activity, Return to fertility, LAM and FP counseling and services | LAM users-supportive counseling including transition, Reinforced education on return to fertility and sexual activity, FP counseling and services | | Transition counseling for LAM users, FP counseling and services, Referrals for CCC as indicated | Reinforcing health messages, addressing any gaps/missed services | FP and birth spacing interval of 18–23 months = 30% reduction in maternal deaths |
| Newborn/Child services | EBF counseling, Education on Return to sexual activity, Return to fertility, LAM and FP counseling and services | Essential baby care, Baby danger signs and management of complications, Immunization, EBF, Physical exam | | Essential baby care, Danger signs and management of illnesses, Immunization (Polio, DPT, Pneumoccocal, Rotavirus), Physical exam, EBF, Cotrimoxazole at 4 week as indicated | Well baby immunisation (Measles) | FP = 50% reduced risk of prematurity and LBW; 60% reduction in child deaths (Lancet paper).  PMTCT goals achieved.  Breastfeeding goals: Non-breastfed children have 4 to 6 times risk of child deaths compared to breastfed (WHO). Reduced disease burden and child mortality from vaccine preventable diseases e.g. measles, tetanus. |

### b: Postnatal care packages for infants after birth: Standard health services and schedules

| **Birth and 48-hour postnatal** | **24 – 48 hours after birth** | **At 1-2 weeks** | **At 4 -6 weeks** | **IMPACT** |
| --- | --- | --- | --- | --- |
| Services | Ensure warmth and put hat on baby. Delay baby’s first bath for the first 24 hours. If pre-term encourage skin-to-skin care. Encourage early initiation of EBF. Tetracycline eye ointment 1%. Vitamin K. Immunization (BCG & Oral Polio). Infant prophylaxis for HIV as indicated. Treatment or refer the infant for complications. Encourage and facilitate birth registration | Vitamin A if not yet given. Immunizations if not yet started. INH prophylaxis as appropriate. Treatment of any complications detected. Referral as appropriate. Birth registration if not yet done | Immunizations (DPT_1_, HeB_1_, Hib_1_, OPV1.) as per schedule, INH prophylaxis as appropriate. Treatment of any complications detected. Referral as appropriate. Early infant diagnosis (EID) for HIV. Management of HIV positive infant. Birth registration if not yet done | Reduce 50% deaths that occur within one day of childbirth; Reduce 30% stillbirths that occur during labour. |
|  | **At 4 – 6 months** | **6 – 7 months** | **7 – 8 months** | **At 8 – 9 months** |
| Services | Vitamin A supplementation. Immunizations as per schedule. INH prophylaxis as appropriate. Treatment of any complications detected. Referral as appropriate. Birth registration if not yet done. Record in Integrated Register and Mother Child booklet | Growth monitoring; Chart weight and height, head to toe examinations, assess danger signs for baby, check immunization status, treatment of any complications, counsel mother on baby danger signs, breastfeeding, complimentary feeding and handwashing of care giver. | Growth monitoring; Chart weight and height, head to toe examinations, assess danger signs for baby, check immunization status, treatment of any complications, counsel mother on baby danger signs, breastfeeding, complimentary feeding and handwashing of care giver. | Growth monitoring; Chart weight and height, head to toe examinations, assess danger signs for baby, check immunization status, treatment of any complications, counsel mother on baby danger signs, breastfeeding, complimentary feeding and handwashing of care giver. |
|  | **At 9-10 months** | **At 10 – 11 months** | **At 11 – 12 months** |  |
|  | Growth monitoring; Chart weight and height, head to toe examinations, assess danger signs for baby, check immunization status, treatment of any complications, counsel mother on baby danger signs, breastfeeding, complimentary feeding and handwashing of care giver. **Measles immunization.** | Growth monitoring; Chart weight and height, head to toe examinations, assess danger signs for baby, check immunization status, treatment of any complications, counsel mother on baby danger signs, breastfeeding, complimentary feeding and handwashing of care giver. | Growth monitoring; Chart weight and height, head to toe examinations, assess danger signs for baby, check immunization status, treatment of any complications, counsel mother on baby danger signs, breastfeeding, complimentary feeding and handwashing of care giver. |  |

**c: Immunization schedule in Kenya**

| **Vaccine** | **Age** | **Remarks** |
| --- | --- | --- |
| BCG, POLIO (OPV O) | At birth dose | Or at first contact with child |
| DPT_1_, HeB_1_, HiB_1_, OPV1 | 6 weeks (1$\frac{1}{2}$months) | Or at first contact with child at that age |
| DPT_2_, HeB_2_, Hib_2_, OPV2 | 10 weeks (2$\frac{1}{2}$ months) | 4 weeks after DPT_1_ and OPV1 can also be given any time after this period, when in contact with the child |
| DPT_3_, HeB_3_, Hib_3_, OPV3 | 14 Weeks (3$\frac{1}{2}$ months) | 4 weeks after DPT_2_ and OPV2 can also be given any time after this period, when in contact with the child |
| Measles | 9 months | May be given between 6 months and 9 months if child is admitted to hospital for any other illness. Repeat at 9 months as per KEPI schedule |

*BCG – Tuberculosis, DPT – Diphtheria, whooping cough and tetanus, OPV – Oral polio vaccine, HeB – Hepatitis B, Hib – Haemophilus influenza
