# Supplementary material for: Conditional cash transfers to retain rural Kenyan women in the continuum of care during pregnancy, birth and the postnatal period: protocol for a cluster randomized controlled trial
Source: Trials. 2019 Mar 1;20:152. doi: 10.1186/s13063-019-3224-8 (PMC6397480; doi:10.1186/s13063-019-3224-8)
Supplement: Supplementary file 2 — Afya secondary data. Information collected on all women who make facility visits in Siaya County, Kenya. (DOCX 36 kb) [file 13063_2019_3224_MOESM2_ESM.docx]

### Additional file 2: Afya Secondary Data. Information collected on all women who make facility visits in Siaya County, Kenya

| ANC REGISTER | MATERNITY REGISTER | POSTNATAL REGISTER |
| --- | --- | --- |
| BACKGROUND | BACKGROUND INFORMATION | REGISTRATION INFORMATION |
| Date of visit | (same as ANC) | Date of visit |
| ANC number | Admission Number | PNC register number |
| 1st visit | Date of Admission | Admission number |
| No. Of visits |  | Full names |
| Name | Diagnosis | Village/Estate |
| Village/Estate | Duration of labour | Age |
| Age | Date of delivery | MATERNITY HISTORY |
| Marital status (married, widowed, single, divorced, seperated) | Time of delivery | Date of delivery |
| LMP | Gestation at birth (weeks) | Place of delivery |
| EDD | Mode of delivery (normal, caesarian, breech, assisted vaginal, seperated) | Mode of delivery |
| Gestation in weeks | Placenta complete (y/n) | STATUS OF BABY |
| Weight (Kg) | Blood loss (in mls) | VITAL SIGNS |
| Blood pressure | Condition after delivery | Temperature |
| **COUNSELLING SERVICES PROVIDED** | Other delivery complications | Pulse |
| Birth plan (y/n) | **BABY** | Blood pressure |
| Danger signs | Sex | POSTNATAL EXAMINATIONS |
| FP | Birth weight (in grams) | Parlor |
| HIV | Live birth/Fresh Still birth/Macerated still birth | Breast |
| Supplement feeding | APGAR score | Uterus |
| Breast care | VDRL/RPR results (for syphillis) | PPH |
| Infant feeding | HIV STATUS | C-section side |
| ITN | ANC | Lochial |
| **LABORATORY, INCLUDING HIV** | Maternity | Episiotomy |
| Haemoglobin | ARV prophylaxis | HIV STATUS |
| RPR/VDRL | HIV status ANC | Prior known status |
| HIV results | HIV status maternity | Tested in PNC (<=72 hours/>72 hours) |
| ART (anti retro virals) eligibility. Assed through: | ARV prophylaxis, mother | PROPHYLAXIS |
| Start of ART in ANC, Date ___ | ARV baby | NVP to baby (y/n) |
| *Prophylaxis; Dispensed ARVs* | CTX to mother (y/n) | CTX baby (y/n) |
| Mother | Vitamin A (y/n) | CTX mother (y/n) |
| NVP for baby (y/n) | Partner HIV councelling & Testing | Partner HIV C & T |
| **SCREENING** | Delivery conducted by (name) | Couple counselled (y/n) |
| TB | Birth notification number | Partner tested in PNC |
| Cervical cancer | Discharge date | Results |
| *Other conditions* | Status of baby at discharge (dead/alive) | Screening for cervical cancer (method, result) |
| Hypertension | Comments | Provided with modern family planning method |
| Diabetes |  | TREATMENT |
| Epilepsy |  | multivitamin |
| Malaria in Pregnancy |  | Haematinic |
| STIs/RTI (syphyllis etc) |  | Referred (yes/no) |
| Others (specify) |  | Remarks |
| **STANDARD TREATMENT** |  |  |
| Deworking (y/n) |  |  |
| IPT 1-3 |  |  |
| TT Dose 1 - 5 |  |  |
| Iron (y/n) |  |  |
| Folic acid (y/n) |  |  |
| Received ITN (y/n) |  |  |
| **ADDITIONAL TREATMENT** |  |  |
| Hypertension |  |  |
| Diabetes |  |  |
| Epilepsy |  |  |
| Malaria in Pregnancy |  |  |
| STIs/RTI |  |  |
| Others (specify) |  |  |
| **PARTNER HIV CARE AND TREATMENT** |  |  |
| Councelled as a couple (y/n) |  |  |
| Test results for partner |  |  |
| Referred for _____ |  |  |
| **REMARKS** |  |  |
