# Supplementary material for: Conditional cash transfers to retain rural Kenyan women in the continuum of care during pregnancy, birth and the postnatal period: protocol for a cluster randomized controlled trial
Source: Trials. 2019 Mar 1;20:152. doi: 10.1186/s13063-019-3224-8 (PMC6397480; doi:10.1186/s13063-019-3224-8)
Supplement: Supplementary file 3 — Annex 11a: Participant’s information sheet part 1, English. (DOCX 14 kb) [file 13063_2019_3224_MOESM3_ESM.docx]

### Annex 11a: Participant’s Information Sheet Part 1, English

AFYA CREDIT INCENTIVE FOR IMPROVED MATERNAL AND CHILD HEALTH OUTCOMES

INFORMATION THAT YOU NEED TO KNOW AS A PARTICIPANT IN THIS RESEARCH

The information provided in this document is to assist in providing detailed information about our research project, and what will be required of you if you agree to enroll as a participant. The information contained here is divided into four sections: why we are doing this study, how you become a study participant and risks and benefits of participating in the study. There is additional information on how the Afya Credit Scheme works, that will be shown to you on a video. The person enrolling you will be available to answer any questions you have after watching the video.

**About this research**

This study is being led by Dr. Caroline Ochieng, a Kenyan researcher who is based at Stockholm Environment Institute, an international organisation with offices in Stockholm and Nairobi. The study is being implemented with a local NGO SWAP that is based in Kisumu and has been working for 10 years in this region on issues of health of women and community. The project is funded by the Bill and Melinda Gates Foundation, a Grant-making foundation that supports initiatives in education, world health and population, and is based in the United States of America.

The reason all these agencies have come together for this work is because we are all concerned about the very high rates of illness and death among pregnant women in Kenya, and would like to explore possible solutions for it. Studies in other regions have shown that these deaths can be prevented if women were to attend all their clinic appointments, give birth in health facilities under skilled attendance and come back for health appointments after birth. But research has also shown that very few women come for all these visits as required due to a variety of reasons.

In this study, we would like to test if the promise of a reward (Afya Credit) at the end of each honored appointment can encourage pregnant women not to miss their health appointments. Research in other countries show that this approach could work, but there is no evidence of this in Kenya. We want to test this system with pregnant women in Siaya County, because the policy makers in this region have indicated that it is one of the areas with low health facility attendance during pregnancy and after birth. We are calling this cash reward system “Afya Credit”.

Because this is a research, we can only test this system in a few individuals that we select, not everybody. The procedures for carrying out this study will be explained to you clearly by the health facility staff that will enroll you in the project if you are willing to be a participant.

**How you enroll in the research**

The project will be carried out in 48 health facilities (dispensaries and health centres) in Siaya County that are selected through a ballot system.

When you attend your normal clinic the nurse will first ask you four questions to check if you are eligible to participate in this study. If you answer yes to the four questions, the nurse will ask you to pick a random card (Afya Card) from a pile of cards, and to touch it on a card reader. The card reader will reveal whether you are in the intervention arm or the control arm of the study. The card you pick determines your study group; the nurse does not know this information until after you have touched your card on the reader.

The nurse will then explain to you all the details about the study, including showing you a short video about the study. You will be free to ask as many questions as you wish. If you are satisfied with all the responses and wish to participate in the study, you will be asked to sign a form stating that the study details have been explained to you, and you are happy to participate. If you are not satisfied and wish to withdraw from the study you will be free to make this choice and will continue to receive your services at the clinic as normal. You will not be penalised for choosing not to participate in the study. Furthermore, even after you have enrolled and signed the forms, you are still free to withdraw at any point.

There is no fee you pay for participating in this study. Every pregnant woman who gives a positive response to the four screening questions has an equal chance of being enrolled in the study. Should you feel that you were discriminated upon during the selection process, or if you have been asked to pay money to participate, report this to us immediately by calling or texting our research staff in SWAP on 0727655426 or the project leader on +46737078583. Call or SMS these numbers if you also feel aggrieved by any of the study procedures, and if you have any questions or concerns about the study that the nurse has not answered to your satisfaction.

**Risks and benefits of participating in the study**

The direct benefits for participation in this study is the Afya Credit reward which will be explained to you in detail. However, even non participants in the project can derive benefits associated with this research in the future. If it is proven that Afya Credit approach can actually work in making women visit health facility as required, we will have meetings with health policy makers in this region and discuss with them if the system can be rolled out to the entire region and countrywide.

There is no foreseen risk of participating in this study, and your participation in it is out of your own free will. The information obtained from you during the study will not be directly linked to you. This will be achieved by identifying you by the number on your Afya card rather than names, so that whoever looks at the information will not be able to trace it back to you.
