# Supplementary material for: Conditional cash transfers to retain rural Kenyan women in the continuum of care during pregnancy, birth and the postnatal period: protocol for a cluster randomized controlled trial
Source: Trials. 2019 Mar 1;20:152. doi: 10.1186/s13063-019-3224-8 (PMC6397480; doi:10.1186/s13063-019-3224-8)
Supplement: Supplementary file 4 — Annex 13a: Informed consent form, English. Annex 14a: Consent to access medical records, English. (ZIP 23 kb) [file 13063_2019_3224_MOESM4_ESM.zip › Additional file 4b Consent for Medical RecordsR1.docx]

### Annex 14a: Consent to Access Medical Records, English

AFYA CREDIT INCENTIVE FOR IMPROVED MATERNAL AND CHILD HEALTH OUTCOMES

CONSENT FOR ABSTRACTION OF MEDICAL RECORDS

I consent to medical information being obtained from my clinic records for the purposes of the Afya study which I have already agreed to participate in. I understand that the information will be treated with utmost confidentiality, that my name will not be listed anywhere on the data extraction forms, and there will be no way the information can be linked back to me. The people extracting the data will have no connection whatsoever to me personally, or to anyone I know.

It has been explained to me and I have understood that this information is to be used only for the purposes of research and not any other purpose. I understand that I have the right to accept or refuse access to all or some of the information in my records without suffering any negative consequences. It has been explained to me that my acceptance or refusal to have my medical data accessed does not affect my enrolment status in the project.

I also understand that I can change my mind at any time about my medical data getting accessed. If I have any concerns about this process, I can reach the Research Coordinator on Tel. 0727655426 or the Principal Investigator on Tel. +46737078583.

| **HEALTH RECORDS FROM ANC REGISTER** | **Consent given (y/n)** | **Signature of participant^*^** |
| --- | --- | --- |
| **Background information** such as my age, where I live, expected delivery data, number of health visits I have made to the clinic. |  |  |
| **Type of health information and services I receive during clinic visits** such as counselling on birth plan, HIV, family planning, pregnancy danger signs, infant feeding. |  |  |
| **Results of my screening tests** such as Malaria, TB, cervical cancer, HIV, STIs, diabetes |  |  |
| **Treatments** I have received such as deworming, tetanus, iron, folic acid, insecticide treated nets, ARV |  |  |
| **Referrals** I have been given for further medical checks and treatments |  |  |

**Sign once (below) if you are giving consent for access to all ANC records. Otherwise sign also in the respective boxes.*

| PARTICIPANT  Name ______________________________  Signature ___________________________  Date _______________________________ | STUDY SUPERVISOR  Name __________________  Signature _______________  Date ___________________ |
| --- | --- |

| **HEALTH RECORDS FROM MATERNITY REGISTER** | **Consent given (y/n)** | **Signature of participant^*^** |
| --- | --- | --- |
| **Background information** such as date of admission and discharge, who attended to me at delivery, date of delivery |  |  |
| **Delivery information** such as duration of labour, gestation at birth, mode of delivery, blood loss at delivery, my health condition after delivery and any delivery complications |  |  |
| **Information about the baby** at delivery such as birth weight, vital signs, health status of the baby at birth |  |  |
| **Test results** for me and my baby such as HIV, STI |  |  |
| **Type of services** I received such as HIV counselling & Testing |  |  |
| **Treatments** I and the baby received such as ARV prophylaxis, vitamin A |  |  |

**Sign once (below) if you are giving consent for access to all maternity records. Otherwise sign also in the respective boxes.*

| PARTICIPANT  Name ______________________________  Signature ___________________________  Date _______________________________ | STUDY SUPERVISOR  Name __________________  Signature _______________  Date ___________________ |
| --- | --- |

| **HEALTH RECORDS FROM POSTNATAL REGISTER** | **Consent given (y/n)** | **Signature of participant^*^** |
| --- | --- | --- |
| **Delivery information** above if it is missing from maternity records |  |  |
| Information on my **health status** and the health status of the baby |  |  |
| **Medical tests** conducted on me and the baby and the rest results |  |  |
| **Treatments** me and the baby have received including malaria, multivitamin, ARV, family planning |  |  |
| Information on **counselling services** I received, whether or not I was counselled with my partner and whether my partner was tested for HIV |  |  |
| Information on whether or not I was **refereed** for further tests or treatments and the referral facility. |  |  |

**Sign once (below) if you are giving consent for access to all postnatal records. Otherwise sign also in the respective boxes.*

| PARTICIPANT  Name ______________________________  Signature ___________________________  Date _______________________________ | STUDY SUPERVISOR  Name __________________  Signature _______________  Date ___________________ |
| --- | --- |
