# Supplementary material for: Conditional cash transfers to retain rural Kenyan women in the continuum of care during pregnancy, birth and the postnatal period: protocol for a cluster randomized controlled trial
Source: Trials. 2019 Mar 1;20:152. doi: 10.1186/s13063-019-3224-8 (PMC6397480; doi:10.1186/s13063-019-3224-8)
Supplement: Supplementary file 4 — Annex 13a: Informed consent form, English. Annex 14a: Consent to access medical records, English. (ZIP 23 kb) [file 13063_2019_3224_MOESM4_ESM.zip › Additional file 4a Informed ConsentR1.docx]

### Annex 13a: Informed consent Form, English

AFYA CREDIT INCENTIVE FOR IMPROVED MATERNAL AND CHILD HEALTH

INFORMED CONSENT FORM

I have freely agreed to participate in this study that is testing if offering cash rewards to pregnant women will make them come for all their health appointments during pregnancy, child birth, and for 12 more months after birth until their children reach 1 year of age**.** It has been explained to me what is expected of me as a participant in each of the two study arms.

The information sheet on the study has been fully read and explained to me, and I have been given an opportunity to ask questions on aspects that I do not understand, for which I have been answered to my satisfaction. I have been informed that if I have any further questions, I should feel free to contact Mr. Aloyce Odhiambo of SWAP Kenya on Tel. 0727655426. I can also contact Dr. Caroline Ochieng, the officer leading the project on phone number +46737078583 or her institution’s office in Nairobi Office on +254 207 224 886.

I understand if I am unable or unwilling to continue with the study at any point, I am free to withdraw without giving a reason. Except for losing the Afya rewards, I will not suffer any other negative consequences as a result withdrawing my participation from the study.

I understand that my name will not be linked with the research materials without my expressed permission.

The contents of this form have been fully explained to me and I agree to take part in this study.

------------------------------------------------------------------------------------------------------

I consent to participate in this study.

________________ _________________ ________________

Participant’s Name Signature/thumbprint Date

(I*N BLOCK LETTERS)*

--------------------------------------------------------------------------------------------------

I have explained and defined in detail the research procedure in which the respondent has consented to participate.

_____________________ _____________________ _______________

ALOYCE ODHIAMBO Signature Date

*_____________________ _____________________ _______________*

*CAROLINE OCHIENG* Signature Date
